# Natural Products in Cardiovascular Diseases: The Potential of Plants from the Allioideae Subfamily (Ex-Alliaceae Family) and Their Sulphur-Containing Compounds

**DOI:** 10.3390/plants11151920

**Published:** 2022-07-25

**Authors:** Jorge M. Alves-Silva, Mónica Zuzarte, Henrique Girão, Lígia Salgueiro

**Affiliations:** 1 Coimbra Institute for Clinical and Biomedical Research, University of Coimbra, 3000-548 Coimbra, Portugal; jmasilva@student.ff.uc.pt (J.M.A.-S.); monica.zuzarte@uc.pt (M.Z.); hmgirao@fmed.uc.pt (H.G.); 2 Faculty of Pharmacy, University of Coimbra, 3000-548 Coimbra, Portugal; 3 Center for Innovative Biomedicine and Biotechnology, University of Coimbra, 3000-548 Coimbra, Portugal; 4 Clinical Academic Centre of Coimbra, University of Coimbra, 3000-075 Coimbra, Portugal; 5 Chemical Process Engineering and Forest Products Research Centre, University of Coimbra, 3030-290 Coimbra, Portugal

**Keywords:** *Allium*, *Tulbaghia*, extracts, sulphur-containing compounds, antiplatelet aggregation, hypertension, diabetes, dyslipidaemia

## Abstract

Cardiovascular diseases (CVDs) are the leading cause of mortality worldwide and, together with associated risk factors such as diabetes, hypertension, and dyslipidaemia, greatly impact patients’ quality of life and health care systems. This burden can be alleviated by fomenting lifestyle modifications and/or resorting to pharmacological approaches. However, due to several side effects, current therapies show low patient compliance, thus compromising their efficacy and enforcing the need to develop more amenable preventive/therapeutic strategies. In this scenario, medicinal and aromatic plants are a potential source of new effective agents. Specifically, plants from the Allioideae subfamily (formerly Alliaceae family), particularly those from the genus *Allium* and *Tulbaghia*, have been extensively used in traditional medicine for the management of several CVDs and associated risk factors, mainly due to the presence of sulphur-containing compounds. Bearing in mind this potential, the present review aims to gather information on traditional uses ascribed to these genera and provide an updated compilation of in vitro and in vivo studies validating these claims as well as clinical trials carried out in the context of CVDs. Furthermore, the effect of isolated sulphur-containing compounds is presented, and whenever possible, the relation between composition and activity and the mechanisms underlying the beneficial effects are pointed out.

## 1. Introduction

Cardiovascular diseases (CVDs) continue to lead mortality rates worldwide [1], accounting for nearly 18 million annual deaths, primarily due to coronary heart disease and stroke [2]. Unfortunately, these numbers tend to increase as several non-modifiable and modifiable risk factors associated with the onset and development of these disorders are also escalating. While non-modifiable risk factors such as aging, gender, genetic predisposition, family history of heart-related problems and ethnicity cannot be altered [3,4,5], modifiable risk factors are changeable. These include hypertension, dyslipidaemia, diabetes, obesity, smoking, alcohol misuse, unhealthy diet, sedentary lifestyle, and psychosocial factors [6] and are recognised as relevant targets to manage CVDs. For example, the INTERHEART case–control study pointed out that 90% of acute myocardial infarction cases are due to these risk factors and that controlling or eliminating them per se could lead to a drastic decrease in CVD mortality [7,8]. Indeed, due to their huge impact on CVDs, these risk factors are included in the World Health Organisation (WHO) target list that aims to reduce their prevalence by 2025 [9]. The negative impact of CVDs is further fuelled by the fact that 60% of patients fail to correctly adhere to the therapeutic regimen [10], mainly due to the cost of CVD therapies [11]. Therefore, new therapeutic interventions and/or preventive strategies with fewer side effects are mandatory, with aromatic and medicinal plants emerging as promising agents to manage both CVDs and associated risk factors. In fact, herbal medicines are relevant sources of bioactive molecules, used by ca. 80% of the world’s population in basic health care [12]. Moreover, many of these medicinal plants have already been used in the treatment of chronic and acute conditions including CVDs [13,14,15,16] and are part of the Mediterranean-style diet with proven beneficial effects on cardiovascular risk factors [17], as pointed out in several meta-analysis and critical reviews [18,19,20,21,22,23]. Interestingly, these effects are associated with the increased consumption of fruit, vegetables, spices, garlic, and onions [24]. Overall, the preventive/therapeutic potential of aromatic and medicinal plants is mainly attributed to the presence of secondary metabolites [25] including phenolic compounds, terpenes, alkaloids, and organosulfur compounds [26]. Organosulfur compounds are widely found in plants from the Allioideae subfamily (ex-Alliaceae family) and, together with extracts or raw bulbs from these plants, are widely reported for their medicinal properties [27]. Therefore, bearing in mind the bioactive potential of these plants, a systematised review gathering information on the effects of sulphur-containing extracts/compounds on major CVD risk factors, namely hypertension and dyslipidaemia/diabetes, is presented. Additionally, whenever reported, the mechanisms underlying the observed effects are referred to and the relation between composition and activity pointed out. To achieve this, a bibliographic search was conducted using Pubmed, Scopus and Google scholar databases, combining the keywords “*Allium*”, “*Tulbaghia*”, “Alliaceae” or “Allioideae” with “cardiovascular”, “diabetes”, “obesity”, “dyslipidaemia”, “hypertension” or “vasorelaxation”. Studies published over the last 20 years that had an available DOI were considered.

## 2. Importance of Allioideae Species in Cardiovascular Diseases

In the following sections, both the relevance and potential of plants from the Allioideae subfamily are described. First, the traditional uses ascribed to these plants in several ethnobotanical surveys is shown in order to highlight their importance in local health care systems. Then, studies validating some of these effects are systematised, considering pre-clinical approaches and clinical trials. The effect of isolated sulphur-containing compounds is also presented, and whenever possible, the relation between composition and activity is discussed, thus opening new avenues for further investigations in the field.

### 2.1. Traditional Uses of Allioideae

A plethora of traditional uses are ascribed to Allioideae plants or plant-based preparations, as summarised in Table 1. Plants’ scientific and common names are included as well as the region of use. In addition, the plant part or preparations used (with reference to the preparation method and posology, when known) and beneficial effects on the cardiovascular system are pointed out. Overall, the majority of the studies focus on the genus *Allium*, with only a few studies reporting the effects of two species from the *Tulbaghia* genus. In traditional preparations, the plant bulb is commonly used (7/11 total studies), with leaves (1/11), aerial parts (1/11) or whole plants (1/11) referred to in much less often. In addition, the use of a combination of plants is frequent and, therefore, this information is also provided. A list of abbreviations, used throughout the table, is provided at the end of the table.

### 2.2. Pre-Clinical Studies Validating the Cardioprotective Effects of Allioideae

Given the importance of Allioideae plants in the management of CVDs and associated risk factors in ethnopharmacological studies, we next compile several pre-clinical studies validating these effects. First, the effect of plants or their extracts is pointed out (Table 2) followed by the effect of isolated sulphur-containing compounds (Table 3) and then clinical trials.

#### 2.2.1. The Effect of Plant Parts or Extracts

In Table 2, studies reporting the beneficial effects of plant parts or extracts is presented with reference to the species name, the plant part/extract used (with reference to the preparation method and concentration), the study model and the main findings regarding the effect observed in the cardiovascular system. Unless stated, a daily administration was used. Studies are grouped considering the cardiovascular disease and/or risk factor assessed with plants organised in alphabetical order of their scientific name. A list of abbreviations, used throughout the table, is provided at the end of the table.

Plants from this subfamily are rich in cysteine sulfoxide derivatives, such as alliin [132], which, by the action of alliinase, are converted into thiosulfinates, e.g., allicin, which in turn are instable and change into organosulfur compounds like ajoene [133]. Thiosulfinates are considered to be the main class of compounds responsible for the biological activities reported for plants from Allioideae subfamily [134]. Accordingly, several studies have assessed the role of alliinase activity on the effect of the extracts. Indeed, the antihypertensive effect of onions (*Allium cepa*) is lost or is much weaker upon boiling [81]. Similarly, the antiplatelet aggregation potential of these bulbs is also compromised, since longer heating times in either a conventional oven or microwave led to a pro-aggregatory effect rather than the expected anti-aggregatory potential [109]. Additionally, with the loss of alliinase activity, the vasorelaxant properties of *Allium sativum,* were abolished in aortic rings pre-contracted with phenylephrine [95]. Similar dependency on alliinase activity was reported for the hypolipidemic activity of *A. sativum* where long heating times or microwave heating compromised this effect [135].

On the other hand, the antidyslipidaemic and antidiabetic effects of *A. sativum* seem to depend on the PI3K/Akt/Nrf2 [87] or IGFIR/PI3K/Akt [67,68] pathways since, upon treatment, activation of these pathways is observed.

In order to better disclose the putative factors underlying the hypolipidemic effect of *A. hookeri*, a metabolomic analysis on the serum of hamsters consuming a high-fat diet and administered *A. hookeri* powder orally was carried out. The authors found 25 putative markers which could explain the lipid-lowering effect of this species, with phosphatidylcholines, lysophosphatidylcholines and lysophosphatidylethanolamines the most common targets. Furthermore, the metabolism for glycerophospholipids was increased in the treated group [57].

#### 2.2.2. The Effect of Isolated Sulphur-Containing Compounds

In this section, the effect of isolated sulphur-containing compounds found in the Allioideae subfamily is presented. Then, a composition–activity relation is discussed in order to bring attention to potential active extracts. Table 3 systematises the main studies performed in these compounds, with the compound name, chemical structure, study model used, and the main findings of the study pointed out. Additionally, whenever reported, the route of administration and concentration used is highlighted. A list of abbreviations, used throughout the table, is provided at the end of the table.

**Table 3:** Effects of sulphur-containing compounds on the cardiovascular system.

Compound	Study Model: Insult or Injury (Route of Administration; Concentration)	Main Findings	Ref.
Ajoene 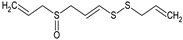	Smooth muscle cells (1–50 μM)	↓ Proliferation, cholesterol biosynthesis	[136]
Allicin 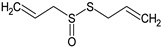	Mice: ApoE-deficient and LDLR-deficient (p.o.; 9 mg/kg)	↓ Atherosclerotic plaque, uptake and degradation of oxLDL by macrophages	[137]
	HUVEC: oxLDL-induced damage (10, 30, 100 μM)	↓ Apoptosis	[138]
	In chemico: Cu^2+^-induced oxidation of LDL from treated ApoE/LDLR-deficient mice (p.o.; 9 mg/kg)	↓ LDL oxidation	[137]
	In chemico: Cu^2+^-induced LDL oxidation (0.1, 1 and 10 mM)	↑ LDL oxidation (at higher doses)	[60]
	Phe-contracted PA rings (0.1, 0.3 and 1.0 µg/mL)	Induced relaxation	[95]
	Rat: SHR (p.o. for 6 weeks; 80 mg/kg on chow)	↓ SBP and TG	[139]
Alliin 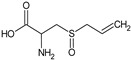	Rat: High fructose (p.o. for 3 weeks; 0.111 and 0.222 mg/kg)	↑ Heart function; ↓ SBP	[88]
	Rat: ISO-induced myocardial infarction (gastric intubation for 35 days; 40 and 80 mg/kg)	↓ CK, CK-MB, LDH, ALT, AST, TC, LDL, VLDL, TG, FFA, PL, MDA levels, HMGR activity; ↑ HDL levels, LCAT activity	[140]
Diallyl disulphide 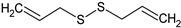	HEPC: In vitro neovasculogenesis (0.1, 1, and 10 μM)	↑ Tube formation, c-kit/PI3K/Akt pathway	[141]
	Rat: Diabetic cardiomyopathy (gavage every other day for 16 days; 40 mg/kg)	↓ Cardiac apoptosis and apoptotic markers dependent of death receptor and mitochondria; ↑ PI3K/Akt pathway	[68]
	HUVEC: Ox-LDL-induced damage (100 and 200 µM)	↑ eNOS phosphorylation at Ser1177, NO and cGMP levels; stabilised eNOS/Cav-1 interaction; ↓ eNOS degradation, proteosome activity	[142]
	HUVEC: Non-stimulated and stimulated (0.2 to 500 µM)	Non-stimulated: ↓ MMP-2 secretion and activity and TIMP-1 secretion Stimulated: ↓ MMP-9 and TIMP-1 secretion	[143]
	In chemico: Isolated xanthine-oxidase activity (5 and 10 µM)	Restored activity in the presence of Cu^2+^	[144]
	In chemico: Cu^2+^ and amphotericin-induced LDL oxidation (5 and 10 µM)	↓ MDA	
	Rat: ISO-induced myocardial necrosis (p.o. for 14 days; 8.94 mg/kg)	↓ HW, LDH, CK-MB, cTnC and systemic inflammation; ↑ SOD and cat	[48]
Diallyl trisulphide 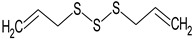	Rat: Diabetic cardiomyopathy (gavage every other day for 16 days; 40 mg/kg)	↓ Cardiac apoptosis	[68]
	HUVEC: Ox-LDL-induced damage (20 and 50 µM)	↑ eNOS phosphorylation at Ser1177, NO and cGMP levels; stabilised eNOS/Cav-1 interaction; ↓ eNOS degradation, proteosome activity	[142]
	Rat: metabolic syndrome (gavage every second day for 3 weeks; 40 mg/kg)	↓ TG, LDL, homocysteine, BG, insulin, MDA, O_2_^2+^, NF-κB, IL-17A, Bax, caspase-3 and -9 mRNA; ↑ HDL, H_2_S, NO_2_^−^, cat, GSH, SOD, cardiac function, eNOS, SOD1/2 and Bcl-2 mRNA	[145]
	HEK293 cells: Whole cell patch clamp (n/a)	↓ IKr and hERG channel trafficking	[146]
	Cardiomyocytes: HG-induced apoptosis (10 μM)	↓ Apoptosis	[147,148]
	Rat: STZ-induced diabetic (i.p. for 14 days; 500 μg/kg)	↑ NO, eNOS proteins and phosphorylation levels, blood perfusion and capillary density	[149]
	HUVEC (1.3, 2.5, 5, and 10 µM)	↓ Tube formation, VEGF2 release and VEGF2R expression	[150]
	HEPC: In vitro neovasculogenesis (0.1, 1, and 10 μM)	In vitro: ↑ tube formation	[141]
	Rat: In vivo neovasculogenesis (gavage for 2 weeks; 10 mg/kg)	In vivo: ↑ new vessels in a xenograft model of neovasculogenesis	
Dimethyl disulphide 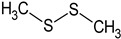	PA: Phe-induced contractions (cumulative doses from 100 nM to 3 μM)	Induced relaxation; ↑ NOS phosphorylation and Ca^2+^ influx to ECs	[84]
S-allylcysteine 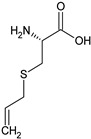	Rat: Acute myocardial infarction (i.p. for 7 days pre-surgery + 2 days post-surgery; 50 mg/kg)	↓ Mortality, infarct size; ↑ CTH activity	[151]
	Cu^2+^-induced LDL oxidation (0.1, 1 and 10 mM)	↓ Oxidation	[60,152]
	Macrophages and HUVEC: oxLDL stimulated (2.5, 5, 10 and 20 mM)	↓ H_2_O_2_ production	[152]
	HUVEC: TNF-α and H_2_O_2_ stimulated (2.5, 5, 10 and 20 mM)	↓ NF-κB activation	
	HUVEC and macrophages: LPS- and IFNγ stimulated (20, 40 and 80 µM)	HUVEC: ↑ eNOS activity, cGMP levels Macrophages: ↓ iNOS activity	[127]
	Rat: ISO-induced myocardial infarction (p.o. every other day for 3 weeks; 13.1 mg/kg and 32.76 mg/kg)	↓ LDH, CK-MB; ↑ heart function; SOD and Cat	[42,43,153]

↑—Increase; ↓—Decrease; Akt—v-Akt Murine thymoma viral oncogene/Protein kinase-B; ALT—Alanine aminotransferase; ApoE—Apoliprotein E; AST—Aspartate aminotransferase; Bax—Bcl-2-associated X protein; Bcl-2—B-cell lymphoma 2; BG—Blood glucose; Cat—Catalase; Cav-1—Caveolin-1; cGMP—Cyclic guanosine monophosphate; CK—Creatine kinase; CK-MB—Creatine kinase muscle/brain isoform; CTH—Cystathionine-γ-lyase; cTnC—Cardiac troponin C; Cu^2+^ —Copper (II); EC—Endothelial cell; eNOS—Endothelial nitric oxide synthase; FFA—Free fatty acid; GSH—Glutathione; H_2_O_2_—Hydrogen peroxide; H_2_S—Hydrogen sulphide; HDL—High-density lipoprotein; HDL—High-density lipoprotein; HEK293—Human embryonic kidney 293 cell; HEPC—Human endothelial progenitor cell; hERG—Human ether-a-go-go-related gene; HG—High glucose; HMGR—3-Hydroxy-3-methylglutaryl-Coenzyme A reductase; HUVEC—Human umbilical cord vein endothelial cell; HW—Heart weight; IFNγ—Interferon gamma; IKr—Delayed rectifier potassium current; IL—Interleukin; i.p.—Intraperitoneal injection; ISO—Isoproterenol; LCAT—Lecithin-cholesterol acyltransferase; LDH—Lactate dehydrogenase; LDL—Low-density lipoprotein; LDLR—Low-density lipoprotein receptor; LPS—Lipopolysaccharide; MMP—Matrix metalloproteinase; mRNA—Messenger RNA; n/a- Not available; NF-κB—Nuclear factor kappa-light-chain-enhancer of activated B cells; NO—Nitric oxide; NO_2_^−^—Nitrite; O_2_^2+^ —Superoxide; oxLDL—Oxidised low-density lipoprotein; PA—Pulmonary artery; Phe- Phenylephrine; PI3K—Phosphoinositide 3-kinase; PL—Phospholipid; p.o.—*Per os* (orally); SBP—Systolic blood pressure; SHR—Spontaneously hypertensive rat; SOD—Superoxide dismutase; STZ—Streptozotocin; TC—Total cholesterol; TG—Triglycerides; TIMP-1—Tissue inhibitor of metalloproteinase-1; TNF-α—Tumour necrosis factor alpha; VEGF2—Vascular endothelial growth factor 2; VEGF2R—Vascular endothelial growth factor 2 receptor; VLDL—Very low-density lipoprotein.

Despite the interest in these compounds and their potential, only one study from those listed in Table 3 focused on the mechanisms of action underlying the observed effects. Indeed, it was shown that allicin reduced oxidised low-density lipoprotein-induced damage by inhibiting apoptosis and decreasing oxidative stress [138].

Although the compounds presented in Table 3 are commonly found in plants from the Allioideae subfamily, there are others that, despite being found in lower amounts, have been assessed for their cardioprotective effect. For example, the antidyslipidaemic effects reported for garlic (*A. sativum*) seem to be due to the capacity of S-allyl cysteine, N-acetyl-S-allyl cysteine, alliin, allixin, and allylmercaptocysteine to suppress low-density lipoprotein oxidation since all these compounds were able to reduce LDL oxidation induced by copper (II) [60]. Additionally, S-methylcysteine sulfoxide in high cholesterol-fed rats, was able to reduce the levels of total cholesterol, triglycerides and phospholipids. Furthermore, this compound reduced the activity of lipoprotein lipase without affecting the activity of other lipogenic proteins, while decreasing the levels of free fatty acids. In addition, the excretion of bile acids and sterols was enhanced in the treated group [154].

Furthermore, the antiplatelet activity of aged garlic extract was related to the presence of *S*-ethylcysteine, *S*-methyl-L-cysteine, *S*-1-proponyl-L-cysteine, since the remaining constituents of the extract (alliin, cycloalliin, *S*-allyl-L-cysteine, *S*-allylmercapto-L-cysteine, and fructosyl-arginine) failed to significantly inhibit platelet aggregation [116]. Moreover, two compounds, sodium n-propyl thiosulfate and sodium 2-propenyl thiosulfate decreased adenosine diphosphate-induced platelet aggregation in both dogs and human blood [155].

Regarding the vascular protective effect of garlic, it seems that allithiamine (vitamin B analogue found in garlic) might play a relevant role. Indeed, the presence of this compound in HUVEC growing in high glucose conditions showed a lower level of advanced glycation end products as well as a lower inflammatory profile when compared to high glucose-only treated cells. In addition, this compound also showed a very potent antioxidant potential [156]. Moreover, 2-vinyl-4H-1,3-dithiin, an organosulfur compound found in macerated garlic oil or in stir-fried garlic, decreased spontaneously hypertensive rat’s vascular smooth muscle cells proliferation and cell migration and arrested cell cycle at G2 phase. Furthermore, it decreased reactive oxygen species production induced by angiotensin II [157]. Also, diallyl disulphide and diallyl trisulphide have been reported for their capacity to induce neovasculogenesis via PI3K/Akt pathway activation [68,141]. In addition, reduction of cell death dependent on death receptor and mitochondria is also reported for both compounds [68]. Furthermore, for diallyl trisulphide, the promotion of neovasculogenesis is also attributed to a decrease in the microRNA 221 [68,141]. This compound also activated Nrf2 via the PI3K/Akt pathway [147] and induced the release of hydrogen sulphide by cystathionine-γ-lyase [148] using in vitro conditions mimicking diabetes. The reported effects for ajoene might be due to its capacity to inhibit protein prenylation, particularly that dependent on protein farnesyltransferase and protein geranylgeranyltransferase type I [136].

Some studies also assessed the activity of synthetic derivatives of naturally occurring sulphur-containing compounds. A study compared the antihypercholesterolaemic properties of diallyl disulphide analogues and showed that all the tested analogues lowered serum and hepatic levels of several lipids, including low-density lipoprotein while increasing those of high-density lipoprotein. The authors suggested that this lipid-lowering effect is due to the modulation of the 3-hydroxy-3-methylglutaryl-CoA reductase activity since a decrease in mRNA levels with a concomitant inactivation of sterol regulatory element-binding protein-2 and cyclic adenosine monophosphate response element-binding protein is observed [158]. Another study assessed the antihypertensive and vasorelaxant properties of five synthetic derivatives of diallyl disulphide. The results showed that all analogues were able to decrease systolic blood pressure in the N^ω^-nitro-L-arginine methyl ester-induced hypertensive animal model. Similarly, all compounds restored the antioxidant defences as observed by an increase in the activity of glutathione peroxidase, glutathione and superoxide dismutase with concomitant decrease in malondialdehyde and protein carbonyl levels. Furthermore, nitric oxide metabolites and cyclic guanosine monophosphate levels were restored by all the analogues, while the activity of angiotensin-converting enzyme was decreased [159].

The effect of sulphur-containing compounds on the pharmacodynamic and pharmacokinetics of other drugs was also assessed. Indeed, it was reported that the oral co-consumption of diallyl trisulphide and nifedipine led to a higher maximum concentration and area under the curve, thus suggesting that the compound might affect the gastrointestinal metabolism of nifedipine, since no effect on the pharmacokinetics was observed when nifedipine was given intravenously [160].

#### 2.2.3. Clinical Trials

The importance of plants from the Allioideae subfamily is also validated by a small number of clinical trials. However, some contradictory results have been reported that may be related to the different doses used, duration of the treatment and/or association with other compounds. For example, in a small placebo-controlled and double-blind trial, firefighters were given four tablets containing 300 mg/table of aged garlic extract and 30 mg/table of coenzyme Q10 for up to 1 year. The results showed that the consumption improved their vascular elasticity and endothelial function [161]. In another study, the consumption of 1200 mg of this extract daily for 4 weeks followed by 4 weeks of washout had no effect on several parameters assessed such as glycated haemoglobin A1c, blood pressure, total cholesterol, triglycerides and high-density lipoprotein, and did not prevent endothelial dysfunction, oxidative stress or inflammation in patients with type 2 diabetes with high cardiovascular risk [162]. Furthermore, the administration of aged garlic extract (250 mg) supplemented with vitamins B12 and B6, folic acid and L-arginine daily for a 12-month period increased the ratio between brown and white epicardial adipose tissues with concomitant increase in the temperature-rebound index, while decreasing homocysteine levels and preventing the progression of coronary artery calcification [163]. In patients with coronary artery calcification and increased cardiovascular disease risk, the consumption of 2400 mg of aged garlic extract daily for 1 year inhibited the progression of the calcification. Regarding secondary outcomes, the extract decreased interleukin-6 levels as well as the glucose levels and blood pressure [164]. The same concentration increased cutaneous microcirculation in diabetic patients, thus suggesting that aged garlic extract might promote wound healing in these patients [165]. Overall, it seems that longer treatment durations (up to 1 year) have better outcomes.

Regarding other extracts, the consumption of 125 mL of red wine extract of onion twice daily for 10 weeks by healthy individuals showed hypocholesterolaemic, antioxidant and anti-inflammatory effects [166]. Additionally, the consumption of 300 mg of *A. sativum* standardised powder for 8 weeks by patients undergoing haemodialysis decreased the absolute values for oxidised low-density lipoprotein and homocysteine. In addition, the powder significantly ameliorated the values of calcium, triglycerides, oxidised low-density lipoprotein and homocysteine [167]. The consumption of quercetin-rich *A. cepa* extract daily for 6 weeks decreased systolic blood pressure in hypertensive individuals when compared to the placebo group [168]. The consumption of *A. cepa* peel extract twice daily for 12 weeks improved the flow-mediated dilation as well as the number of circulating endothelial progenitor cells in healthy overweight and obese patients. Indeed, the rate of patients with endothelial dysfunction decreased from 26% to 9% after extract administration [169].

Concerning CVD risk factors, some studies have been performed, such as the Tehran Lipid and Glucose study that assessed the effect of dietary consumption of *A. sativum* and *A. cepa* in cardiometabolic risk factors (body mass index, waist circumference, systolic blood pressure, diastolic blood pressure, fasting plasma glucose, triglycerides-to-high-density lipoprotein ratio, insulin, creatinine, estimated glomerular filtration rate and creatinine clearance) for 6 years. The results showed that high consumption of these vegetables led to a 64% reduction in CVD outcomes, as well as a lower incidence of chronic kidney disease and hypertension while no association was made with type 2 diabetes. Furthermore, it improved TG levels and creatine clearance [170].

Although the majority of the reported clinical trials are conducted using a small cohort of patients and are usually single-centre studies, they highlight the potential of plants from the Allioideae subfamily in the management of CVDs and associated risk factors. Nevertheless, these effects should be validated in more complete clinical trials with access to bigger multicentre cohorts to account for the genetic polymorphisms which impact the activity of drug metabolising enzymes leading to altered pharmacokinetics [171].

Drug interactions between conventional drugs or between these and herbal medicines are common, with both beneficial and detrimental effects reported. For example, the consumption of capsules containing 0.5 g of *A. macrostemon* bulb extract powder (three times a day) for eight weeks by patients undergoing baseline therapy for unstable angina, led to lower oxidised low-density lipoprotein and plasminogen activator inhibitor-1 level, while increasing plasminogen activity [172]. Moreover, in patients undergoing simvastatin therapy, supplementation with fenugreek and garlic for 8 weeks significantly reduced total cholesterol, triglycerides, non-high-density lipoprotein and low-density lipoprotein levels and increased those of high-density lipoprotein [173]. On the other hand, care must be taken with antiplatelet drugs, particularly warfarin and aspirin, as a simultaneous consumption of garlic or onion with these drugs can increase the risk of bleeding [174,175]. This interaction is attributed to their capacity to decrease platelet adhesion and aggregation, by inhibiting plasminogen activating factor and fibrinogen receptors and by decreasing thromboxane X_2_ synthesis [176]. In addition, garlic consumption is known to inhibit CYP3A4, the enzyme responsible for warfarin metabolism [175].

## 3. Final Remarks

The present review sheds light on the potential of plants from the Allioideae subfamily in the management of CVDs and associated risk factors. Traditional uses of some of these species are widely recognised, with garlic (*Allium sativum*) and onions (*Allium cepa*) being the most common. Additionally, pre-clinical studies and clinical trials validating their beneficial potential are frequent, thus confirming their importance. Nevertheless, other species such as *A. jacquermontii*, *A. rotundum* and *Tulbaghia alliacea*, despite being used in traditional remedies in some regions, lack scientific validation while other plants have undergone clinical trials but with no beneficial effects on the cardiovascular system.

Regarding CVD risk factors, plants from the Allioideae subfamily showed promising antiplatelet aggregation, antidiabetic, and dyslipidaemic effects, and were able to exert protection against atherosclerotic events.

Overall, we gathered information on both the tapped and untapped potential of plants belonging to the Allioideae subfamily, by highlighting scientific gaps as well as well-validated effects that pave the way for the development of new preventive/therapeutic approaches for CVDs.

## Figures and Tables

**Table 1:** Traditional uses ascribed to plants from the Allioideae subfamily.

Scientific Name (Common Name)	Region of Use	Plant Part or Preparation (Mode of Administration; Posology)	Cardiovascular Disease/Risk Factor (Observed Effect)	Ref.
*Allium ampeloprasum* L. (wild leek)	Suva planina, Serbia	Raw aerial parts (oral)	Diabetes	[28]
*Allium cepa* L. (onion)	Serra de Mariola, Spain	Whole plant (oral)	Hypertension	[29]
	Beni Mellal, Morocco	Raw bulb (oral)	Diabetes	[30]
	Gabon	Bulb (oral)	Diabetes, hypertension	[31]
	Edo, Nigeria	Mined and blended bulb mixed with honey (oral; one tablespoonful twice a day)	Hypertension	[32]
		Bulb maceration—soaked in water with *Vernonia amygdalina* and *Zingiber officinale* for 5 days (oral; one cup twice a day)		
		Bulb decoction with snail water and *Capsicum frutescens*, add small salt and filter (oral; one small cup twice daily)		
		Bulb concoction with *Viscum album*, *Persea americana*, *Ocimum gratissimum* added with *Elaeis guineensis* kernel oil and boiled for 10 min (oral; drink as a soup)		
	Tamil Nadu, India	Bulb mixed with buttermilk (oral)	Cardiovascular disease	[33]
		Bulb boiled in milk, sugar from *Borassus flabellifer* added (oral; once a day in the evening)		
		Raw bulb, (oral; daily before eating for 45 days)	Dyslipidaemia	
		Raw bulb (oral; daily in the morning for 20 days)		
		Juice from bulbs (oral; 25 mL in the morning for two weeks)	Obesity	
		Bulb decoction with *Macrotyloma uniflorum*, *Zingiber officinale* and honey (oral; daily in the morning for 30 days)		
*Allium jacquemontii* Kunth.	Dir, Pakistan	Raw bulb, (oral; for 3 weeks)	Hypertension	[34]
*Allium rotundum* L.	Aladaglar, Turkey	Raw bulb (oral)	Hypertension (regulates blood pressure)	[35]
*Allium sativum* L. (garlic)	Eastern Cape, South Africa	Not referred	Diabetes	[36]
	Gabon	Bulb maceration	Diabetes, dyslipidaemia	[31]
	Suva planina, Serbia	3 peeled bulbs with 3 chopped lemons in 1 L of hot water for 12 h) (oral; 1 cup a day for 40 days)	Dyslipidaemia (decreases TG), hypertension (improves blood circulation)	[28]
	Togo	Bulb decoction with *Khaya senegalensis* bark (oral)	Diabetes	[37]
		Bulb maceration with honey (oral)	Hypertension	
		Bulb maceration with *Parkia biglobosa* (oral)		
		Bulb powder containing *Lippia multiflora*, *Stachytarpheta angustifolia* and *Persea americana* (oral)		
	Edo, Nigeria	Bulb maceration—soaked with guava, *Vernonia amygdalina* in water for 5 days (oral; half a cup daily)	Hypertension	[32]
		Bulb decoction combined with *Allium cepa* and boiled in water (oral; half a cup twice a day)		
		Bulb decoction combined with *Allium cepa* and *Zingiber officinale* and boiled in water (oral; one cup twice a day)		
		Bulb decoction with *Cocos nucifera* boiled for 3 days (oral; half a cup twice a day)		
		Bulb infusion after pounding with *Carica papaya* (oral; one small cup 3 times a day)		
		Bulb decoction with *Musanga cecropoides, Talinum triangulare, Carica papaya* boiled in water (oral; half a cup twice a day)		
		Bulb decoction with *Hunteria umbelleta, Sida acuta* and potash in cold water (oral; one cup a day)		
	Western Anti-Atlas, Morocco	Raw bulb (oral)	Diabetes	[38]
	Tamil Nadu, India	Boiled bulb with *Zingiber officinale* rhizome and added milk (oral; twice a day)	Cardiovascular disease	[33]
		Bulb cooked with *Foeniculum vulgare* in milk (oral; daily in the morning until cure)		
		Bulb cooked with *Trachyspermum ammi* in milk (oral; daily in the morning until cure)		
		Bulb boiled in water, and added milk (oral; daily in the evening)	Hypertension	
		Bulb boiled in milk (oral; daily in the evening for a month)	Obesity	
		Bulb as a food supplement with *Moringa oleifera* (oral; twice a week at lunch)	Hypertension	
		Bulb syrup with *Citrus limon* juice, *Zingiber officinale* rhizome, *Malus pumila* cider vinegar and honey (oral; 10 mL twice a day)	Cardiovascular disease, obesity	
		Bulb syrup with *Citrus limon* juice, *Zingiber officinale* rhizome, *Malus pumila* cider vinegar and honey (oral; 15 mL twice a day after meals)	Cardiovascular disease, dyslipidaemia	
		Bulb paste with *Coriandrum sativum*, *Solanum torvum* and *Zingiber officinale*, consumed with honey (oral; once a day in the morning)	Cardiovascular disease	
		Bulb combined with honey (oral; 5 mL once a day in the morning)		
		Bulb combined with honey (oral; 5 mL twice a day for a month)	Dyslipidaemia	
		Bulb combined with honey (oral; 10 mL twice a day)	Hypertension	
		Bulb jam with sugar from *Borassus flabellifer* and oil from *Sesamum indicum* (oral; 10 g twice a day for 45 days)	Dyslipidaemia	
	Tamil Nadu, India	Bulb jam with sugar from *Borassus flabellifer* (oral; 20 g twice a day till cure)	Obesity	
		Bulb jam with sugar from *Borassus flabellifer* and oil from *Sesamum indicum* (oral; 10 g twice a day)	Hypertension	
		Bulb powder with *Cinnamomum verum*, *Piper cubeba* and *Vitis vinifera* (oral; 2–3 g once a day in the evening)	Dyslipidaemia	
		Bulb gravy with *Arachis hypogea*, *Cissus quadrangularis*, *Murraya koenigii*, *Tamarindus indica* and clarified butter (oral; twice a week until cure)		
	Beni Mellal, Morocco	Raw bulb (oral)	Diabetes	[30]
*Allium ursinum* L. (wild garlic)	Suva planina, Serbia	Leaf tincture diluted in a small glass of water (oral; 10 drops 3 times a day before meals)	Hypertension, hypercholesterolemia (lowers blood cholesterol)	[28]
*Tulbaghia alliacea* L.	Eastern Cape, South Africa	Not referred	Diabetes	[36]
*Tulbaghia violaceae* Harv.				

TG—triglycerides.

**Table 2:** Effects of plant parts/extracts from the Allioideae on the cardiovascular system.

Plant Species	Plant Part or Extract Used (Preparation; Concentration)	Study Model: Insult or Injury	Main Findings	Ref.
Ischaemic injury/Myocardial infarction				
*Allium cepa*	Aqueous extract (i.v. 30 min before injury; 0.1, 0.3 and 1 g/kg)	Rat: Brain ischaemia	↓ Brain edema; prevented ZO-1 and occludin disruption; ↑ Cat and GPx; ↓ MDA	[39]
	Methanolic extract (0.01, 0.05 and 0.1 g/mL)	Cardiomyoblasts (H9c2): Hypoxia	↓ ROS production, mitochondrial membrane depolarisation, cytochrome c and caspase-3 release	[40]
	Methanolic extract (p.o. 14 days before injury; 0.1, 1 and 10 g/Kg)	Rat: Ischaemic injury	↓ Infarct area, apoptotic cell death and MDA	
*Allium macrostemon*	Decoction with bulbs and *Trichosanthes kirilowii* (gavage for 4 weeks; 1.14, 2.27 and 4.53 g/Kg)	Rat: LAD ligation-induced infarction	↓ HW/BW, LV/BW, systemic inflammation, myocardial fibrosis, and collagen I and III expressions; ↓ TGFβ1, TGFβ2 and Smad 2/3 expression; ↑ Smad7 expression	[41]
*Allium sativum*	Aged garlic extract (p.o. for 3 weeks; 2 and 5 mL/Kg)	Rat: ISO-induced myocardial infarction	↑ Heart function, SOD and Cat; ↓ LDH, CK-MB and MDA	[42,43]
	Homogenate (p.o. for 30 days; 125, 250 and 500 mg/kg)		↑ SOD and cat; ↓ LDH, CK-MB and structural changes	[44]
	Raw homogenate (p.o. 30 days before injury; 125, 250 and 500 mg/kg)		↓ MDA, LDH and structural changes	[45]
		Rat: I/R	↓ MDA and structural changes; ↑ SOD, cat, GSH and GPx	[46]
	Black garlic extract (gavage for 4 weeks; 300 mg/kg)		↑ HO-1	[47]
	Raw garlic extract (gavage for 4 weeks; 300 mg/kg)		↑ HO-1 and eNOS	
	Garlic oil (intragastric for 14 days, 100 mg/kg)	Rat: ISO-induced myocardial necrosis	↓ HW, LDH, CK-MB, cTnC and systemic inflammation; ↑ SOD and cat	[48]
*Allium ursinum*	Methanolic extract (p.o. 28 days before injury in drinking water; 125, 250, and 500 mg/kg)	Rat: I/R	↑ Cardiac function and antioxidant system	[49]
*Tulbaghia violacea*	Methanolic extract (intragastric for 30 days before injury; 60 mg/kg)	Rat: ISO-induced myocardial infarction	↓ CK, CK-MB, LDH and MDA; ↑ LV function, SOD and GSH	[50]
Dyslipidaemia/Diabetes/Metabolic syndrome				
*Allium cepa*	Aqueous extract (p.o. for 4 weeks; 0.5, 1.5 and 4.5 g/kg)	Rat: HFD-induced hyperlipidaemia	↓ TC, LDL, MDA, lipid droplets in liver, foam cell accumulation and HMG-CoA; ↑ HDL, SOD and LDLR	[51]
*Allium cepa* var. *destiny* and var. *cavalier*	Raw onion (p.o. for 6 weeks; 16 and 40 g/kg)		↓ TC, glucose, LDL, HDL, TG, erythrocyte number and haemoglobin; ↑ white blood cell number	[52]
*Allium elburzense*	Hydroalcoholic extract (intragastric for 7 days, 100, 200, and 400 mg/kg)	Rat: DEX-induced diabetes	↓ TG, TC, LDL, MDA and liver steatosis; ↑ HDL	[53]
*Allium eriophyllum*	Hydroalcoholic extract (gavage for 4 weeks; 30 and 100 mg/kg)	Rat: T2DM + Hypertension	↓ SBP, BG, CK-MB, infarct size and coronary resistance; ↑ SOD, GSR	[54]
*Allium hirtifolium*	Ethyl acetate fraction from hydroalcoholic extract (gavage for 4 weeks; 5 mg/kg)	Rat: STZ-induced diabetes	↓ BG; ↑ LVDP, HR, RPP and +dp/dt	[55]
*Allium hookeri*	Powder (p.o. for 4 weeks; 3% and 5% in chow)	Rat: HFD-induced obesity	↓ BW, BW gain, adipose tissue, TG, TC, LDL, AI, cardiac risk factor, LDH, AST and ALP	[56]
	Powder (p.o. for 13 weeks; 0.2 g/Kg)	Hamster: HFD-induced obesity	↓ TG, TC and LDL	[57]
	Hydroalcoholic extract (p.o. for 4 weeks; 200 and 400 mg/kg in chow)	Mice: HFD-induced obesity	↓ liver and adipose tissue weight, TG, TC, LDL, AI, AST and ALT; ↑ HDL	[58]
*Allium sativum*	Aged garlic extract (intra-abdominal injection every 12 h for one month; 125 mg/kg)	Rat: Metabolic syndrome	↓ TG, insulin, leptin, AGE, SBP and MDA; ↑ GSH, and GPx; restored vascular and cardiac function	[59]
	Aged garlic extract (1, 2.5 and 5 g/L)	HUVEC: oxLDL	↓ LDH release and cell damage	[60]
		In chemico: Cu^2+^-induced LDL oxidation	↓ Cu^2+^-induced LDL oxidation	
	Aged garlic extract (p.o. for 12 or 24 weeks; 3% in chow)	Mice: ApoE^−/−^	↓ Atherosclerotic lesions, TC, TG and CD11b^+^ cells in spleen	[61]
	Fresh homogenate (intragastric for 41 days; 100 mg/kg)	Pregnant rat: High cholesterol diet	On mothers: ↓ systemic inflammation, disruption of mitochondrial network, infiltration of foam cells, TC, TG, LDL and CK On offspring: ↓ abnormalities and abortions	[62]
	Homogenate and raw garlic (p.o. for 100 weeks; 0.5% in chow)	Rat: High cholesterol diet	Homogenate: ↓ TC, LDL and TG Raw garlic: ↓ TC, LDL and TG; ↑ excretion of TG and TC	[63]
	Aged garlic extract (p.o. for 56 days; 500 mg/kg)	Rat: STZ-induced diabetes	↓ Glucose, CK, LDH and AGER gene expression; ↑ Mn-SOD	[64]
	Raw garlic (p.o. for 4 weeks; 250 mg/kg)		↑ Cat, SOD, SIRT3 activity, TFAM and PGC-1α mRNA; ↓ ROS	[65]
	Black garlic extract (p.o. for one month; 250 mg/kg)	Rat: High fat/sucrose diet	↓ Calory intake, BW, TG, LDL, insulin, leptin and leptin receptor, pro-inflammatory genes; induced vasorelaxation	[66]
	Garlic oil (p.o. for 8 weeks; 1% in chow)	Hamster: High cholesterol	↓ Cardiac apoptosis and apoptotic markers; ↑ IGFR/PI3K/Akt pathway	[67]
	Garlic oil (gavage daily for 16 days; 100 mg/kg)	Rat: Diabetic cardiomyopathy	↓ Cardiac apoptosis and apoptotic markers dependent of death receptor and mitochondria; ↑ IGFR/PI3K/Akt pathway	[68]
	Aqueous extract (5 mg/mL added to the blood collection tube)	Human: Healthy individuals	↓ TC and TG	[69]
	Aqueous extract (i.p. for 8 weeks; 100 mg/kg)	Rat: STZ-induced diabetes	↓ STZ-induced vasoconstriction	[70,71,72]
	Aqueous extract (p.o. for 16 weeks; 100 mg/kg)		↓ Coronary arterioles thickening and BG; ↑ aortic/coronary blood flow	[73]
	Aqueous extract (gavage for 28 days; 2500 and 500 mg/kg)	Rat: Obese and insulin resistant	↓ Insulin, BG, and lipid levels; ↑ cardiac function and mitochondrial homeostasis	[74]
	High pressure garlic extract (p.o. for 5 weeks; 2% in chow)	Rat: High-fat diet	↓ Plasma and hepatic LDL and TG; ↑ plasma HDL, hepatic mRNA ApoA1, ABCA1 and LCAT	[75]
	Bulb powder (gavage for 28 days; 200 mg/kg)	Rat: STZ/Nicotinamide-induced diabetes	↓ Hyperglycaemia, dyslipidaemia, AI and MDA; ↑ Insulin production, GSH activity	[76]
	Powder (p.o. for 35 days; 300 mg/kg)	Rabbit: HC-induced atherogenesis	↓ Neointima formation, cholesterol, TG, PL and collagen accumulation; ↓ TG, TC, PL blood levels; ↓ AI	[77]
	Powder (n/a)	Rat: In vivo Fe^2+^-induced LDL oxidation	↓ LDL oxidation and oxLDL mobility; ↓ MDA hepatic, serum and heart levels	[78]
*Allium ursinum*	Leaf lyophilizate (p.o. for 8 weeks; 2% in chow)	Rabbit: Hypercholesterolaemic	↑ Heart function in vivo and ex vivo; ↑ HO-1; ↓ TC, TG, ApoB and atherosclerotic lesions	[79]
*Tulbaghia violacea*	Methanolic extract (p.o. for 2 weeks; 0.25 and 0.50 g/kg)	Rat: Atherogenic diet	↓ TG, TC, LDL, VLDL, MDA, fibrinogen, LDH, AST, ALT, bilirubin, creatinine, and fatty streak plaques; ↑ HDL, SOD, cat, and NO	[80]
Hypertension/Vasorelaxation				
*Allium cepa*	Raw onion (p.o. for 3 weeks; 5% in chow)	Rat: L-NAME-induced hypertension	↓ SBP and TBARS; ↑ NO metabolites excretion	[81]
*Allium fistulosum*	Raw or boiled juice (cumulative doses from 3 × 10^−5^ to 4 × 10^−3^ g/mL)	Aortic rings: NE precontracted	Raw juice: Induced relaxation Boiled juice: ↑ EDCF	[82]
	Raw green part (p.o. for 4 weeks, 5% in chow)	Rat: HFD-induced hypertension	↓ SBP, O_2_^2−^ and NOX activity; ↑ NO levels	[83]
*Allium macrostemon*	Volatile extract (cumulative doses from 0.01% to 0.1%)	Pulmonary arteries: Phe contracted	Induced relaxation; ↑ NOS phosphorylation and Ca^2+^ influx to ECs	[84]
*Allium sativum*	Aged garlic extract (gavage for 12 weeks; 2 g/kg)	Rat: Dahl salt-sensitive hypertensive	↓ LVEDP, pressure half-time, interstitial fibrosis, LV mass and SBP	[85]
	Aged garlic extract (cumulative doses from 0.001 to 1%)	Aortic rings: NE-contracted	Induced relaxation in a dose-dependent manner	[86]
	Fresh homogenate (p.o. for 8 weeks; 250 mg/kg)	Rat: High fructose	↓ LVH, NF-κB and oxidative stress; ↑ cat, GSH, GPx, and Nrf2	[87]
	Homogenate (p.o. for 3 weeks; 125 and 250 mg/kg)		↓ SBP, HR, TC, TG, glucose, LDH, CK-MB; ↑ SOD, cat and heart function	[88]
	Raw garlic (p.o. 1 day or 3 weeks before MCT injection + 3 weeks; 1% in chow)	Rat: MCT-induced PH	↓ RVSP, RVH, vasoconstriction in CEC; induced relaxation	[89]
	Garlic juice (cumulative doses from 1 to 50 μg/mL)	Aortic rings: Phe contracted	Induced relaxation in a dose-dependent manner	[90]
	Aqueous extract (p.o. for 4 weeks; 50 mg/kg)	Rat: 2-kindey-1-clip hypertension	↓ SBP and ACE activity	[91]
	100% methanol fraction from a methanolic extract (cumulative doses from 30 to 750 µg/mL)	Aortic rings	Precontracted with KCl or Phe: Induced relaxation Pre-treatment with the fraction: Prevented contraction evoked by KCl or Phe	[92]
	Aqueous extract (0.045 mg/mL)	Aortic rings: NE-contracted	Prolonged relaxation induced by GSNO; Inhibited chloride channels	[93]
	Aqueous extract (cumulative doses from 3 to 500 μg/mL)	Pulmonary arteries	Normoxia: Induced dose-dependent relaxation Hypoxia: Inhibited the transient relaxation and sustained contraction elicited by hypoxia ↓ ET-1 induced contractions	[94]
	Aqueous and 5% ethanol extracts (cumulative doses from 1 to 500 μg/mL)		↓ Phe-induced contractions; ↑ ACh-induced relaxation	[95]
	Aqueous and ethanol extract (non-cumulative doses from 0.1 to 3 mg/L)	Atria: Spontaneously or EPI-induced contraction	Negative inotropic and chronotropic effect	[96]
*Allium ursinum*	Leaf lyophilizate (p.o. for 8 weeks; 2% in chow)	Rat: MCT-induced PH	↑ RV function and PDE5 activity; ↓ Medial thickness of PA	[97]
*Tulbaghia violacea*	Methanolic extract (i.p. for 7 weeks; 50 mg/kg)	Rat: Dahl salt-sensitive hypertensive	↓ SBP; ↑ [Na] in urine and AT-1a receptor levels	[98]
Protection against cardiotoxic compounds				
*Allium cepa*	Raw juice (intragastric intubation for 14 days; 1 mL)	Rat: DOX-induced cardiotoxicity	↓ Apoptotic cells; ↓ CK, CK-MB, LDH, cTn1 and MDA levels; ↑ SOD, GSH, GPx	[99]
	Raw juice (intragastric intubation for 14 days; 1 mL)	Rat: DOX-induced endothelial dysfunction	↓ Apoptotic cells; ↓ MDA levels; ↑ GSH	[100]
		Rat: Cd-induced cardiotoxicity	↓ Apoptotic cells; ↓ CK, CK-MB, LDH, cTnT and MDA levels; ↑ SOD, GSH, GPx	[101]
	Raw juice (intragastric intubation for 8 weeks; 1 mL/100 g BW)		↓ TC, TG, LDL, albumin and MDA; ↑ HDL and SOD	[102]
*Allium sativum*	Aged garlic extract (1000 µg)	Rat: DOX-induced cardiomyocyte apoptosis	↓ p53 activation, and caspase-3 activity; ↑ 8-isoprostane levels	[103]
	Aged garlic extract (p.o. for 6 days before DOX; 2860 mg/kg)	Mice: DOX-induced cardiotoxicity	↑ survivability, and tumour uptake of DOX	[103]
	Aged garlic extract (p.o. for 28 days; 250 mg/kg)		↓ LDH, CK and MDA	[104]
	Homogenate (p.o. days; 250 and 500 mg/kg)	Rat: Adriamycin-induced cardiotoxicity	↑ SOD, GPx, and cat; ↓ MDA, TNF-α accumulation	[105]
	Aqueous extract (p.o. for 3 weeks; 250 mg/kg)	Rat: Gentamycin-induced renal failure	↑ Renal function, BW, HW/BW, cardiac Na^+^/K^+^-ATPase activity, and antioxidant capacity; ↓ BP, LDH, CK-MB, MDA	[106]
*Allium ursinum*	Water and methanolic extracts (4 h pre-treatment; 50 μg/mL)	Cardiomyoblasts (H9c2): DOX-induced toxicity	Water: ↓ intracellular and mitochondrial ROS and cell death induced by DOX Methanolic: ↓ intracellular and mitochondrial ROS	[107]
Antiplatelet aggregation				
*Allium ampeloprasum*	Raw juice (n/a)	Human: Platelet aggregation in whole blood	↓ Platelet aggregation (IC_50_ = 114.9 and 117.3 mg/mL)	[108]
*Allium ascalonicum*			↓ Platelet aggregation (IC_50_ = 6.9 and 30.9 mg/mL)	
*Allium cepa*	Heated extract (n/a)	Human: Platelet-rich plasma	↓ Platelet aggregation, which is lost with higher heating times or microwave heating	[109]
	Peel aqueous extract (50, 100 and 500 μg/mL)	Rat: Collagen-induced platelet aggregation	↓ Platelet aggregation, [Ca^2+^]_i_, TXA_2_; ↑ cAMP	[110]
	Methanolic extract and methanolic fractions (0.5, 1, 3 and 5 mg/mL)		↓ Platelet aggregation	[111]
	Raw juice (n/a)	Human: Platelet aggregation in whole blood	↓ Platelet aggregation (IC_50_ = 46.7 and 116.7 mg/mL)	[108]
	Raw juice (i.v. after CFR induction; 0.09 ± 0.01 mL/kg)	Dog: Chronic platelet-mediated thrombosis	↓ Platelet aggregation	[112]
	Raw homogenate (intragastric after CFR induction; 2 g/kg)			
	Raw juice (1, 10 and 100 mL/L)	Human and dog: In vitro platelet aggregation	↓ Platelet aggregation in both blood type, stronger effect on dog	
*Allium fistulosum*	Raw juice (n/a)	Human: Platelet aggregation in whole blood	↓ Platelet aggregation (IC_50_ = 113.8 and 113.2 mg/mL)	[108]
	Raw juice (p.o. for 4 weeks; 2 g/Kg)	Rat	↓ SBP, platelet adhesion to fibrinogen, platelet aggregation and thromboxane release; ↑ bleeding time, cAMP and 6-keto prostacyclin F_1α_	[113]
	Raw or boiled juice (0–4 mg/mL)	Human: ADP-induced aggregation	Raw juice: ↓ [Ca^2+^]_i_ and thromboxane production; ↑ cAMP levels Boiled juice: ↑ [Ca^2+^]_i_ and thromboxane production; induced morphological changes	[114]
*Allium sativum*	Aged garlic extract (3.12 to 12.5%)	Human: Fibrinogen- and ADP-induced platelet aggregation	↓ Platelet adhesion to fibrinogen; Prevented platelet conformational changes induced by ADP; ↑ cAMP	[115]
	Aged garlic extract (0.78–25%)	Human: ADP-induced platelet aggregation	↓ Platelet aggregation and [Ca^2+^]_i_	[116,117]
		Human: ADP aggregated PRP	Induced platelet disaggregation	[116]
	Aged garlic extract (p.o. for 7 or 14 days; 1, 2 or 5 g/kg)	Rat: Healthy fed AGE	↓ Platelet aggregation after 14 days without prolonging bleeding time; ↑ extracellular ATP, TXB_2_ and ↓ phosphorylation of ERK, p38 and JNK after collagen treatment	[118]
	Aged garlic extract (p.o. for 13 weeks; 5 mL)	Human: ADP-induced platelet (13 days pre-treatment)	↓ % of aggregated platelets and the initial rate of aggregation	[119]
	Aged garlic extract (0.19–6.25%)	Human: ADP-induced platelet aggregation	↓ Platelet aggregation; ↑ cGMP and cAMP which were inhibited by ODQ and SQ22536	[120]
	Garlic juice (n/a)	Human: Platelet aggregation in whole blood	↓ Platelet aggregation (IC_50_ = 3.2 and 4.0 mg/mL)	[108]
	Aqueous and alcoholic extract (n/a)	Human: Platelet-rich plasma	Aqueous: ↓ ADP-induced aggregation Alcoholic: ↓ ADP-, AA-, EPI-induced aggregation	[121]
	Odourless powder (p.o. for 2 weeks; 1 g/kg in chow)	Rat: In situ loop	↓ Thrombus formation	[122]
*Allium schoenoprasum*	Raw juice (n/a)	Human: Platelet aggregation in whole blood	↓ Platelet aggregation (IC_50_ = 45.4 and 50.1 mg/mL)	[108]
*Allium ursinum*	Aqueous extract (n/a)	Rat: Platelet aggregation	↓ ADP-, collagen-, AA- and EPI-induced aggregation	[121]
Other activities				
*Allium ampeloprasum*	Aqueous extract (0.045 mg/mL)	NO release from S-nitrosoglutathione	Induced NO release	[93]
*Allium ascalonicum*	Ethyl acetate fraction from a hydroethanolic extract (500 and 800 ng/mL)	HUVEC: Angiogenesis Chorioallantoic membrane assay	Promoted angiogenesis	[123]
*Allium cepa*	Aqueous extract (0.045 mg/mL)	NO release from S-nitrosoglutathione	Induced NO release	[93]
*Allium sativum*	Aged garlic extract (1–4 mg/mL)	HUVEC	↑ HO-1, GCLM and Nrf2 activation	[124]
	Aged garlic extract (p.o. for 6 weeks; 4% in chow)	Rat: Folate-deficient diet	↓ Homocysteine total, protein-bound and free levels	[125]
	Chloroform extract of aged black garlic (30 min before treatment; 30 μg/mL)	HUVEC: TNF-α	↓ ROS, NF-κB activation, VCAM-1 mRNA and protein expression and THP-1 adhesion to HUVEC	[126]
	Aqueous extract (0.045 mg/mL)	NO release from S-nitrosoglutathione	Induced NO release	[93]
	Aqueous extract (0.2–1.0%)	Macrophages/HUVEC: LPS- and IFNγ stimulated	Macrophages: ↓ iNOS expression HUVEC: ↑ eNOS activity and cGMP levels	[127]
	Garlic skin or flesh extract (1, 2.5 and 5 µL/mL)	Cardiomyocyte: NE-induced hypertrophy	↓ Cell hypertrophy, cell death, apoptosis, and oxidative stress	[128]
	Aqueous extract (p.o. for 5 weeks; 800 µg/Kg)	Rabbit: Vascular restenosis	↓ Myointimal hyperplasia	[129]
	Hydroalcoholic extract (1, 10, 50, and 100 μg/mL)	Mice: LPS-stimulated heart	↓ PGE_2_ and 8-iso-PGF_2α_ levels; ↓ COX2, IL-6 and NF-κB mRNA	[130]
	Aqueous fraction of garlic powder (4 days before treatment; 0.25–4.0 mg/mL)	CAEC: IL-1α	↓ ICAM-1, VCAM-1 and monocyte adhesion to ECs	[131]

↑—Increase; ↓—Decrease; +dp/dt—Ratio of pressure change in the ventricular cavity during the isovolaemic contraction period; 8-iso-PGF_2α_—8-iso-Prostaglandin F2α; AA—Arachidonic acid; ABCA1–ATP-binding cassette transporter; ACE—Angiotensin-converting enzyme; Ach—Acetylcholine; ADP–Adenine diphosphate; AGE—Advanced glycation end products; AGER—Advanced glycation end products receptor; AI—Atherogenic index; Akt—v-Akt Murine thymoma viral oncogene/Protein kinase-B; ALP—Alkaline phosphatase; ALT—Alanine aminotransferase; ApoA1—Apolipoprotein A1; ApoB—Apolipoprotein B; ApoE—Apolipoprotein E; AST—Aspartate aminotransferase; AT-1a—Type 1A angiotensin II receptor; ATP—Adenosine triphosphate; BG—Blood glucose; BW—Body weight; Ca^2+^—Calcium; CAEC—Coronary artery endothelial cells; cAMP—Cyclic adenosine monophosphate; Cat—Catalase; Cd—Cadmium; CEC—Coronary endothelial cells; cGMP—Cyclic guanosine monophosphate; CK—Creatine kinase; CK-MB—Creatine kinase—muscle/brain isoform; COX2—Cyclooxygenase-2; CRF—Cyclic flow reduction cTn1—Cardiac troponin T; Cu^2+^—Copper (II); DEX—Dexamethasone; DOX—Doxorubicin; EC—Endothelial cell; EDCF—eEndothelium-derived contracting factor; eNOS—Endothelial nitric oxide synthase; EPI—Epinephrine; ERK—Extracellular signal-regulated kinases; ET-1—Endothelin-1; Fe^2+^—Iron (II); GCLM—Glutamate-cysteine ligase modifier subunit; GPx—Glutathione peroxidase; GSH—Glutathione; GSNO—S-Nitrosoglutathione; GSR—Glutathione reductase;; C—High cholesterol; HDL—High-density lipoprotein; HFD—High-fat diet; HMG-CoA—β-Hydroxy β-methylglutaryl-Coenzyme A; HO-1—Hemeoxygenase-1; HR—Heart rate; HUVEC—Human umbilical cord vein endothelial cell; HW/BW—Heart weight/body weight ratio; I/R—Ischaemia/Reperfusion; IC_50—_Concentration needed to inhibit 50% of the enzyme activity; ICAM-1– Intercellular adhesion molecule 1; IFNγ—Interferon gamma; IGFR—Insulin-like growth factor 1 receptor; i.p.—Intraperitoneal injection; IL-1α—Interleukin-1 alpha; IL-6—Interleukin-6; iNOS—Inducible nitric oxide synthase; ISO—Isoproterenol; JNK—c-Jun N-terminal kinase; KCl—Potassium chloride; LAD—Left anterior descending; LCAT—Lecithin-cholesterol acyltransferase; LDH—Lactate dehydrogenase; LDL—Low-density lipoprotein; LDLR—Low-density lipoprotein receptor; L-NAME—N^ω^-nitro-L-arginine methyl ester; LPS—Lipopolysaccharide; LV—Left ventricle; LV/BW—Left ventricle weight/body weight ratio; LVDP—Left ventricle diastolic pressure; LVEDP—Left ventricle end-diastole pressure; LVH—Left ventricle hypertrophy; MCT—Monocrotaline; MDA—Malondialdehyde; Mn-SOD—Manganese superoxide dismutase; mRNA—Messenger RNA; n/a—Not available; Na—Sodium; NE—Norepinephrine; NF-κB—Nuclear factor kappa-light-chain-enhancer of activated B cells; NO—Nitric oxide; NOX—Dihydronicotinamide-adenine dinucleotide phosphate oxidase; Nrf2—Nuclear factor erythroid 2-related factor 2; O_2_^2^—Superoxide; ODQ—[1H-[1,2,4]oxadiazolo-[4, 3-a]quinoxalin-1-one]; oxLDL—Oxidised low-density lipoprotein; p38–p38 Mitogen-activated protein kinases; PA—Pulmonary artery; PDE5—phosphodiesterase type 5; PGC-1α—Peroxisome proliferator-activated receptor-gamma coactivator-1alpha; PGE_2—_Prostaglandin E2; PH—Pulmonary hypertension; Phe—Phenylephrine; PI3K—Phosphoinositide 3-kinase; PL—Phospholipid; p.o.—*Per os* (orally); PRP—Platelet-rich plasma; ROS—Reactive oxygen species; RPP—Rate pressure product; RV—Right ventricle; RVH—Right ventricle hypertrophy; RVSP—Right ventricle systolic pressure: SBP—Systolic blood pressure; SIRT3—Sirtuin 3; SOD—Superoxide dismutase; SQ22536—Inhibitor of adenylyl cyclase; STZ—Streptozotocin; T2DM—Type 2 diabetes mellitus; TBARS—Thiobarbituric acid reactive substances; TC—Total cholesterol; TFAM—Mitochondrial transcription factor A; TG—Triglycerides; TGF—Transforming growth factor; THP-1—Spontaneously immortalised monocyte-like cell line; TNF-α—Tumour necrosis factor alpha; TXA_2_—Thromboxane A2; TXB_2_—Thromboxane B2; VCAM-1—Vascular cell adhesion protein 1; VLDL—Very low-density lipoprotein; ZO-1—Zonula occludens-1.

## Data Availability

Not applicable.
